# Zika Virus: What Have We Learnt Since the Start of the Recent Epidemic?

**DOI:** 10.3389/fmicb.2017.01554

**Published:** 2017-08-22

**Authors:** Juan-Carlos Saiz, Miguel A. Martín-Acebes, Rubén Bueno-Marí, Oscar D. Salomón, Luis C. Villamil-Jiménez, Jorg Heukelbach, Carlos H. Alencar, Paul K. Armstrong, Tania M. Ortiga-Carvalho, Rosalia Mendez-Otero, Paulo H. Rosado-de-Castro, Pedro M. Pimentel-Coelho

**Affiliations:** ^1^Department of Biotechnology, Instituto Nacional de Investigación y Tecnología Agraria y Alimentaria Madrid, Spain; ^2^Departamento de Investigación y Desarrollo (I+D), Laboratorios Lokímica Valencia, Spain; ^3^Instituto Nacional de Medicina Tropical Puerto Iguazú, Argentina; ^4^Grupo de Epidemiología y Salud Pública, Universidad de La Salle Bogota, Colombia; ^5^Department of Community Health, School of Medicine, Federal University of Ceará Fortaleza, Brazil; ^6^College of Public Health, Medical and Veterinary Sciences, Division of Tropical Health and Medicine, James Cook University, Townsville QLD, Australia; ^7^Communicable Disease Control Directorate, Western Australia Department of Health, Perth WA, Australia; ^8^Instituto de Biofísica Carlos Chagas Filho, Universidade Federal do Rio de Janeiro Rio de Janeiro, Brazil; ^9^Instituto de Ciências Biomédicas, Universidade Federal do Rio de Janeiro Rio de Janeiro, Brazil; ^10^Instituto D’Or de Pesquisa e Ensino Rio de Janeiro, Brazil

**Keywords:** flavivirus, epidemiology, microcephaly, Guillain–Barré syndrome, antivirals

## Abstract

Zika is a viral disease transmitted mainly by mosquitoes of the genus *Aedes.* In recent years, it has expanded geographically, changing from an endemic mosquito-borne disease across equatorial Asia and Africa, to an epidemic disease causing large outbreaks in several areas of the world. With the recent Zika virus (ZIKV) outbreaks in the Americas, the disease has become a focus of attention of public health agencies and of the international research community, especially due to an association with neurological disorders in adults and to the severe neurological and ophthalmological abnormalities found in fetuses and newborns of mothers exposed to ZIKV during pregnancy. A large number of studies have been published in the last 3 years, revealing the structure of the virus, how it is transmitted and how it affects human cells. Many different animal models have been developed, which recapitulate several features of ZIKV disease and its neurological consequences. Moreover, several vaccine candidates are now in active preclinical development, and three of them have already entered phase I clinical trials. Likewise, many different compounds targeting viral and cellular components are being tested in *in vitro* and in experimental animal models. This review aims to discuss the current state of this rapidly growing literature from a multidisciplinary perspective, as well as to present an overview of the public health response to Zika and of the perspectives for the prevention and treatment of this disease.

## Introduction

Since the beginning of the 21st century, a number of infectious disease threats have emerged that are deemed to be such a risk as to demand a global response. Most have been respiratory viral diseases – severe acute respiratory syndrome (2003), avian influenza in humans (2005), A(H1N1) pandemic influenza (2009), and Middle East respiratory syndrome coronavirus (MERS-CoV) (2012 onward). Ebola virus disease, transmitted to others by direct contact, bucked that trend when a large outbreak in several West African countries claimed over 11,000 lives from 2014 to 2015. Very few would have predicted the most recent infectious disease emergency would involve a vector-borne virus, Zika virus (ZIKV), causing congenital malformations and other neurological disorders.

ZIKV is a flavivirus (*Flaviviridae* family) transmitted by mosquitoes. The virus has been isolated from many mosquito species, although it seems that the vectors of the natural transmission cycle are mosquitoes of the genus *Aedes* (Diagne et al., 2015). As any other flavivirus, the viral genome is composed of a single-stranded RNA molecule of positive polarity about 10 kb in length that encodes a single open reading frame (ORF) flanked by two untranslated regions at both ends (Kuno and Chang, 2007).

ZIKV was first isolated in 1947 from the serum of a febrile sentinel monkey in the Zika Forest, hence its name, and 1 year later from *Aedes africanus* mosquitoes caught in the same forest (Dick et al., 1952). Since then, it was confined to Africa until it first detection in Asia in the 1980s. Subsequently, human outbreaks were reported in 2007 in the Micronesia and in 2013 in the French Polynesia (Saiz et al., 2016). However, ZIKV was an almost neglected pathogen until its recent introduction into the Americas.

It is not the direct effect that ZIKV has on those infected that is the main concern, as the vast majority will be either asymptomatic or else experience a relatively mild illness and an uneventful recovery. Rather, it is the sequelae of infection– Guillain–Barré syndrome (GBS) and microcephaly and other congenital malformations – that cause the morbidity and mortality associated with the infection. As a result, the World Health Organization (WHO) declared a public health emergency of international concern (PHEIC) (WHO, 2016e), elements of which were later integrated into risk assessments by the European Centre for Disease Prevention and Control (ECDC, 2016).

This review discusses several aspects of the biology, epidemiology, transmission and health consequences of ZIKV infection, including findings from *in vitro* and *in vivo* models. Disease control measures, such as vaccine development and the public health response to ZIKV outbreaks, are also reviewed.

## Epidemiology

The emergence of new pathogens has been the reality and a prominent feature of the 21st century. It constitutes a global challenge to public health, especially in developing countries. Arboviruses such as Dengue virus (DENV), Chikungunya and ZIKV are paradigmatic examples of such a statement.

ZIKV virus is a flavivirus first discovered in 1947 in the Zika forest of Uganda, in a captive sentinel rhesus monkey during a yellow fever (YF) surveillance disease activity (Dick, 1953). In 1952, the presence of human cases was demonstrated by a mouse protection test in the sera of indigenous residents of Uganda and Tanganika (Smithburn, 1952). During 1958, the isolation of two strains of the virus were made in mosquitoes (*Aedes africanus*) in Lungo forest (Weinbren and Williams, 1958). The virus was also detected during the decades of 1960–1980 in sentinel Rhesus monkeys and on mosquitoes (mainly the genus *Aedes*) in other countries across equatorial Africa. Sporadic human cases of a mild disease were reported (Saluzzo et al., 1981).

A number of serological studies in the 1950s provided some evidence that ZIKV was widespread throughout Asia (Wikan and Smith, 2016). The presence of ZIKV in Asia was confirmed in 1969 when the virus was isolated from an *Aedes aegypti* mosquito in Malaysia (Marchette et al., 1969).

In April 2007, ZIKV spread its usual geographic range and was detected outside Africa and Asia for the first time when an outbreak occurred on Yap Island in the South Western Pacific Ocean, as an emerging pathogen (Hayes, 2009). Sera from acutely ill patients were sent to the Centers for Disease Control and Prevention (CDC) Arbovirus Diagnostic and Reference Laboratory in Fort Collins, Colorado, where 10 of 71 samples (14%) were found positive for the virus, as they contained ZIKV RNA according to reverse-transcriptase-polymerase-chain-reaction (RT-PCR) assay (Hayes, 2009). It has been found that the attack rates of ZIKV infection were higher among females than males and among older persons than younger persons. In contrast, the prevalence of IgM antibody against ZIKV was higher in male participants (possibly due to their greater exposure to mosquitoes) and was relatively equally distributed across age groups (Hayes, 2009).

Although wind-blown mosquitoes can travel distances of several hundred kilometers over the open ocean, introduction of the virus by travel or trade involving an infected person or an infected mosquito is considered the most likely source of the Yap Island outbreak, especially because no monkeys were present on the island during the outbreak (Hayes, 2009; Kindhauser et al., 2016).

The fact that ZIKV caused such a widespread outbreak on Yap Island, numbering more than 100 confirmed and probable cases, is striking. The absence of any evidence that viral mutation could explain changes in the epidemiological behavior of the virus has led to several other explanations being postulated, including a lack of population immunity; that means that regular exposure to infection by populations in Africa and Asia may have prevented large outbreaks in those regions, such as those seen on the Pacific Islands and in the Americas (Kindhauser et al., 2016).

Another possible reason for this change may lay on the probable under-reporting, which could explain missing reports of previous outbreaks, especially due to the clinical similarities of mild illnesses associated with ZIKV, DENV, and Chikungunya infections, and the frequent co-circulation of all three viruses (Paixao et al., 2016).

Since 2008, the availability of information about the virus has increased, including data on epidemiology, causal associations, and possible sexual transmission. Clinical and serologic evidence suggested that a US-American scientist contracted ZIKV infections while working in Senegal in 2008, and then transmitted this arbovirus to his wife after his return home. Direct contact was implicated as the transmission route, most likely as a sexually transmitted infection (Foy et al., 2011). Additional evidence was found during a ZIKV outbreak in French Polynesia (2013): the virus was isolated from the semen of a patient in Tahiti that sought treatment for hematospermia (Musso et al., 2015).

Retrospective surveys identified an unusual and heterogeneous cluster of congenital brain malformations and brainstem dysfunction in fetuses and newborns over a limited period following a ZIKV epidemic in French Polynesia (Besnard et al., 2016), prompting the conclusion of causal association (Araujo et al., 2016).

In 2016, ZIKV spread rapidly throughout the Americas after its initial appearance in Brazil in May 2015. In 2016, 48 countries and territories in the Americas had reported more than 532,000 suspected cases of Zika, including 175,063 confirmed cases. In addition, 22 countries and territories reported 2,439 cases of a congenital syndrome associated with Zika. Five countries had reported sexually transmitted Zika cases (The Pan American Health Organization [PAHO]/World Health Organization [WHO], 2015; Ikejezie et al., 2017).

In Brazil, the most heavily affected country, a very rapid dispersion of ZIKV was identified, mainly in the Northeast region. This area has the lowest vaccine coverage for YF and notified 65.7% of all cases (De Goes Cavalcanti et al., 2016). Only from March to September 2016, which is considered the highest transmission period in Brazil, as evidenced by a large number of dengue cases observed over years, there was an incidence rate of ZIKV infections of 69.22 cases/100,000 newborns, a mortality coefficient of 5.37 deaths/100,000 newborns and a case fatality rate of 7,750 deaths/100,000 cases. The highest incidence and mortality were found in the Northeast region with 201 cases/100,000 newborns and 4 deaths/100,000 newborns, respectively (SVS/MS, 2017).

The rapid spread of ZIKV disease in the Americas was aided by the lack of effective control of vectors of other arboviruses like DENV and Chikungunya, in several countries of Latin America (Rodriguez-Morales, 2015). For decades, vector control programs have failed, due to the adaptation capacity and biological efficiency of the vectors (Al-Abdely, 2016). Additional challenges for population-based surveillance of ZIKV disease include remote locations, porous borders with neighboring countries, and underreporting by health care providers, all of which can limit the ability to ascertain all cases of ZIKV disease. Underreporting of cases would be particularly marked if they occur in areas that are not considered to be at risk for ZIKV disease, such as those at elevations of more than 2,000 m above sea level (Pacheco et al., 2016).

**FIGURE 1:**
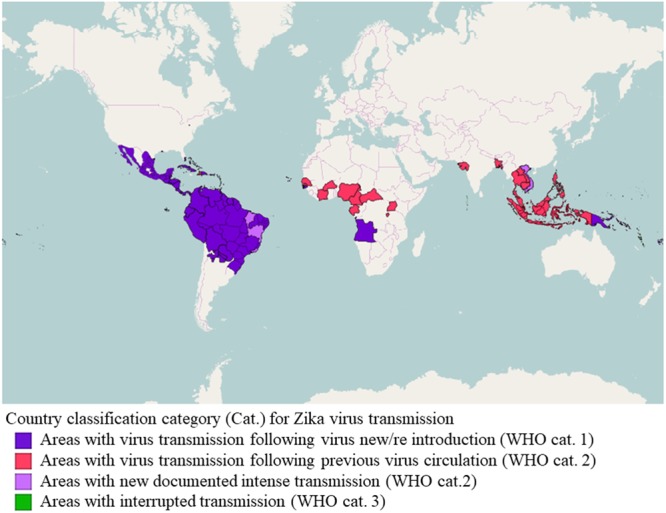
Classification of countries and territories regarding vector-borne Zika virus transmission. Map of the current Zika virus transmission based on the European Center for Disease Prevention and Control (ECDC) adaptation of the World Health Organization (WHO)’s Zika virus country classification scheme (https://ecdc.europa.eu/en/publications-data/current-zika-virus-transmission-list-countries-ecdc-adaptation-whos-zika-virus - accessed 16 July 2017). The map was generated using GADM database of Global Administrative Areas shapefiles (http://www.gadm.org/) and Openlayers plugin within QGIS 2.18.9 (Development Team, 2017, available at http://www.qgis.org/en/site).

To summarize, the global emergence (**Figure 1**) of certain arboviruses, such as ZIKV, that in the past were considered to be restricted to specific geographical areas and to cause a mild disease of sporadic behavior, indicates the importance of such diseases to epidemiology and public health, from the perspective of adaptation potential, certain and probable vectors, surveillance and control, and also communication, interdisciplinary investigation and intersectoral cooperation. In other words, “new threats from infectious diseases may emerge from unexpected places, and we need strategies in place that we can roll out to rapidly gain an understanding of the transmission, pathogenesis, and control of previously little-known pathogens to protect global public health” (Lessler et al., 2016).

Maybe it is time to reconsider research horizons, and to pay attention to the integral circle of host, agent, environment and vector. Health promotion approaches are still valid, and can be an alternative for the global reality of health inequities and weakening of public services (Caprara and Ridde, 2016).

## Biology of the ZIKV

### Molecular Classification and Phylogeny

**FIGURE 2:**
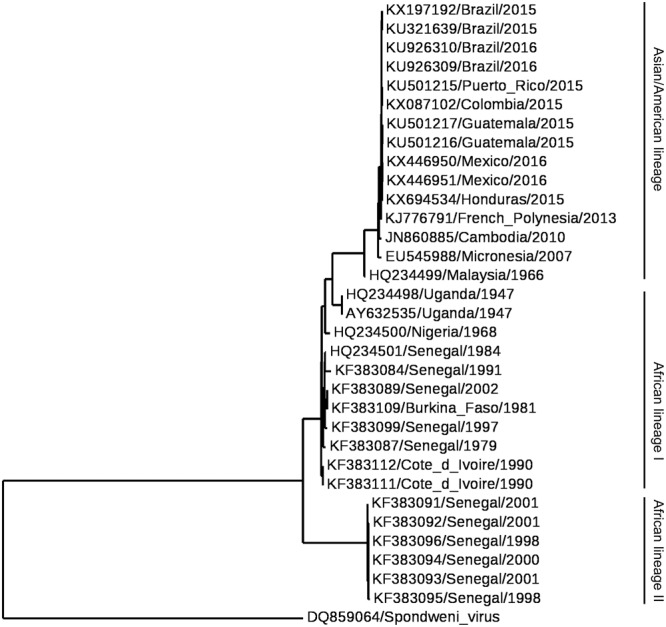
Phylogram of Zika virus (ZIKV). The phylogenetic tree was based on the sequence of NS5. Multiple alignment was performed using MUSCLE (Edgar, 2004) and the tree was constructed by the Maximum Likelihood method using PhyML (Guindon et al., 2010) and Phylogeny.fr (Dereeper et al., 2008). Spondweni virus was included as the outgroup control.

Historically, ZIKV has been classified into the Spondweni serogroup, genus *Flavivirus* (*Flaviviridae*), which includes two species: ZIKV and Spondweni virus (Casals, 1957). Further molecular classifications confirmed these relationships between both species (Kuno et al., 1998; Haddow et al., 2012). Nowadays, ZIKV isolates can be grouped into two or three major lineages (**Figure 2**). These lineages correspond to the African lineage, the Asian lineage (that includes the American strains) and a neglected lineage circulating in Africa (designated African II) that would constitute a sister group to both African (which should be renamed to African I) and Asian lineages previously identified (Faye et al., 2014; Gong et al., 2016; Wang L. et al., 2016; Li Y. et al., 2017). All these phylogenetic analyses indicate that ZIKV originated from Africa and then spread to Asia, Pacific islands, and throughout the Americas. The introduction of ZIKV into the Americas most probably occurred by a single introduction of an Asian strain of ZIKV between May and December 2013 (Faria et al., 2016). Remarkably, despite the genetic differences between ZIKV strains, the antigenic relationships between strains support the existence of a single viral serotype which may be of crucial importance for the design of ZIKV vaccines (Dowd et al., 2016a).

### Genome

**FIGURE 3:**
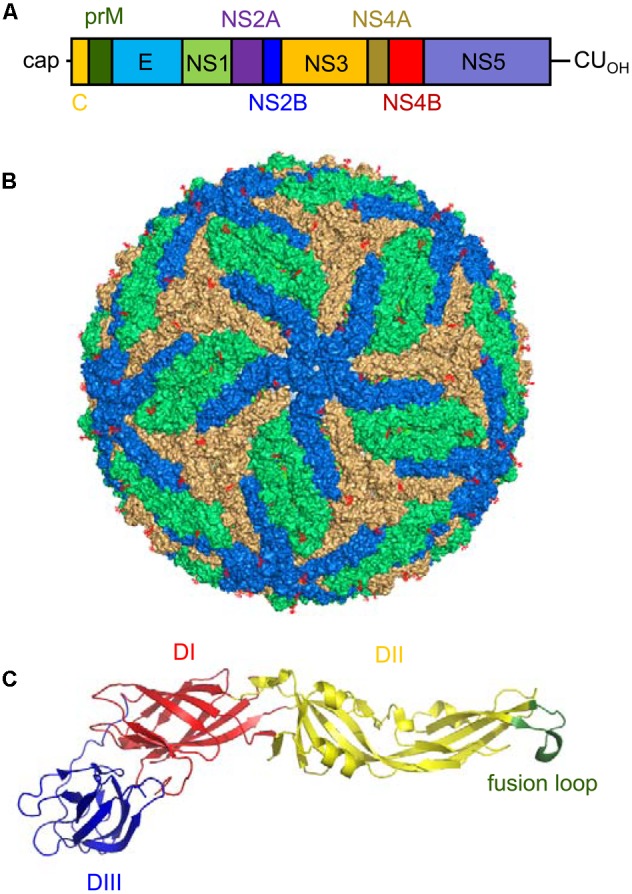
Genomic organization, virion structure, and organization of the E glycoprotein of Zika virus (ZIKV). **(A)** Schematic view of the genomic organization of ZIKV. The single open reading frame (ORF; boxes) that encodes both structural (C-prM/M and E) and non-structural (NS) proteins (NS1, NS2A, NS2B, NS3, NS4A, NS4B, and NS5) is flanked by two untranslated regions (UTRs). Notice the presence of a 5′ cap and the lack of a poly(A) tail at the 3′ end of the genome. **(B)** Surface representation of a ZIKV mature particle. The E monomers are colored in blue, orange and green to facilitate the interpretation of their distribution. Image was produced using the cryo-electron microscopy data available (Protein Data Bank entry 5IRE). **(C)** Structure of a monomer of the soluble ectodomain of E glycoprotein of ZIKV. The ribbon diagram was based on the atomic coordinates solved by X-ray crystallography (Protein Data Bank entry 5JHM). DI in red, DII in yellow, and DIII in blue. Fusion loop is highlighted in green.

The viral genome is composed of a single-stranded RNA molecule of positive polarity about 10 kb in length (Kuno and Chang, 2007). In a similar manner to cellular mRNAs, ZIKV genome includes a cap structure at its 5′ end, but in contrast to cellular mRNAs, ZIKV genome lacks a 3′ poly(A) tract and ends with CU_OH_ (**Figure 3A**). The genome contains a single ORF flanked by two untranslated regions located at the 5′ and 3′ ends of the genome (Kuno and Chang, 2007). ZIKV genome also contains three conserved sequences (CS 1 to CS3) that may mediate genome cyclization between 5′ and 3′ terminal regions of the genome. Notably, the organization of the CS in the 3′ end of ZIKV is different from that of other mosquito-borne flaviviruses (Kuno and Chang, 2007). Besides genomic RNA, it has been described that due to the presence of a multi-pseudoknot structure in the genomic RNA that confounds a cellular exonuclease, ZIKV-infection also produces subgenomic flaviviral RNAs (sfRNAs) within infected cells that play relevant roles in innate immunity evasion and viral pathogenesis (Akiyama et al., 2016; Donald et al., 2016).

### Proteins

The polyprotein encoded by the single ORF in ZIKV is cleaved by cellular and viral proteases into 10 mature proteins (three structural and seven non-structural proteins) (**Figure 3A**). The sequence of the cleavage sites of ZIKV follows the patterns established for other mosquito-borne viruses (Kuno and Chang, 2007). The three structural proteins [the capsid (C), premembrane/membrane (prM/M), and envelope (E) proteins] participate in the assembly of the virions. The C protein conform the core of the virions together with the RNA. The E protein should mediate the binding to the cellular receptor of the virus, promotes the fusion of the virions with the target membranes, and constitutes the main target for the induction of antibodies (Dai et al., 2016; Stettler et al., 2016; Wang Q. et al., 2016; Zhang C. et al., 2016). The E protein is *N*-glycosylated at Asn 154 in most ZIKV strains. This glycosylation is important for particle protein expression and secretion, viral packaging, and infectivity (Mossenta et al., 2017). The cleavage of prM into M protein promotes the maturation of the virions from “spiky” immature particles to “smooth” mature particles (Prasad et al., 2017). In a similar way to that described for other flaviviruses (Martin-Acebes and Saiz, 2012), the seven non-structural (NS) proteins (NS1, NS2A, NS2B, NS3, NS4A, NS4B, and NS5) should account for a variety of functions that go from replication of viral RNA, morphogenesis of viral particles, induction of membrane rearrangements, and viral factory development to modulation of host immune response. The NS1 protein, whose structure has been resolved (Song et al., 2016; Xu X. et al., 2016), participates in flaviviral replication and exhibits immunomodulatory activities. Since NS1 protein is secreted from infected cells, it largely induces antibodies within infected hosts that can be suitable for diagnostics purposes (Dai et al., 2016; Steinhagen et al., 2016). Regarding NS2A, to our knowledge, there are no specific studies addressing the function of this protein in ZIKV. On the other hand, NS3 is a trypsin-like serine protease involved in polyprotein processing (Gruba et al., 2016; Lei et al., 2016) that also exhibits helicase activity, which plays a pivotal role in viral RNA replication enabling RNA unwinding (Tian et al., 2016). Apart from its involvement in viral polyprotein processing, NS3 can cleave cellular factors such as FAM134B, hence modulating the autophagic response within ZIKV-infected cells (Lennemann and Coyne, 2017). NS2B acts as a cofactor necessary for the activity of this protein, and the crystal structure of NS2B-NS3 complex has been resolved under different circumstances (Lei et al., 2016; Phoo et al., 2016; Zhang C. et al., 2016). Due to the relevance of NS2B-NS3 function in the ZIKV life cycle, the search for inhibitors of the enzymatic activity of this complex is at the front line of antiviral discovery against ZIKV (Cao et al., 2016; Sahoo et al., 2016; Lee et al., 2017; Rut et al., 2017). NS4A and NS4B deregulate Akt-mTOR signaling to inhibit neurogenesis and induce autophagy (Liang et al., 2016). In addition, the expression of NS4A has been also related to activation of the cellular stress pathway involving Tor1 and type 2A phosphatase activator Tip41 (Li G. et al., 2017). NS5 is the viral RNA-dependent RNA polymerase that is in charge of genome replication constituting a major target for antiviral design (Lu et al., 2017; Xu et al., 2017). Furthermore, the analysis of the structure of the methyltransferase domain of NS5, which is responsible for capping the 5′ end of the viral genomic RNA, also provides new opportunities for the design of antiviral compounds (Coloma et al., 2016; Stephen et al., 2016; Zhang C. et al., 2016; Coutard et al., 2017; Zhou et al., 2017). Besides its function in genome replication and capping, NS5 from ZIKV also contributes to viral multiplication by inhibiting interferon signaling (Grant et al., 2016). Although a great effort has been performed to decipher the structure and function of some of the ZIKV NS proteins, many issues still remain to be analyzed, as the more detailed knowledge of their function would probably provide valuable information about the pathogenesis of ZIKV, and would also greatly contribute to the development of antiviral strategies against this pathogen.

### Virion

Early filtration studies suggested that the size of ZIKV particles was about 30 to 45 nm in diameter (Dick, 1952). Further transmission electron microscopy showed that the virions were spherical particles with an overall diameter of 40 to 43 nm displaying a central electron dense core being 28 to 30 nm in diameter (Hamel et al., 2015). Nowadays, cryo-electron microscopy reconstructions of mature virions have provided a detailed view of the structure and organization of mature ZIKV particles (Kostyuchenko et al., 2016; Sirohi et al., 2016). The internal core of the particle is composed of the genomic RNA molecule complexed with multiple copies of the capsid (C) protein, enclosed within a lipid membrane derived from the endoplasmic reticulum of the host cell. The E and M proteins (180 copies of each protein) are anchored to this lipid membrane via their transmembrane regions. Since the M protein is a small protein hidden under the E protein layer, the outer layer of the viral particles consists of an icosahedral protein shell basically composed of the E protein (**Figure 3B**). This outer shell exhibits the characteristic herringbone structure in the virion similar to that of other flaviviruses. The E proteins are arranged as 90 anti-parallel homodimers, with three dimers lying parallel to each other forming a raft. The ectodomain of the E protein that protrudes from the lipid bilayer is organized in three different domains: DI, DII, and DIII (**Figure 3C**). DI acts as a bridge between DII and DIII. The tip of DII contains the hydrophobic fusion loop that interacts with cellular membranes for viral fusion (Kostyuchenko et al., 2016; Sirohi et al., 2016). DIII is the target for most neutralizing antibodies, whereas antibodies against DI/DII are poorly neutralizing (Stettler et al., 2016; Zhao et al., 2016). As mentioned above, the E protein is glycosylated at Asn 154 in most ZIKV strains, and this glycan protrudes from the surface of the particle. Remarkably, the region surrounding the glycosylation site structurally greatly differs from other related flaviviruses (Kostyuchenko et al., 2016; Sirohi et al., 2016). Also, in contrast to that described for DENV, ZIKV particles are structurally stable even when incubated at 40°C (Kostyuchenko et al., 2016). Although it has been proposed that this thermal stability may have implications for virus survival in body fluids such as saliva or semen (Kostyuchenko et al., 2016), other studies discard that the unique pathobiology of ZIKV may be only the cause of its thermal stability (Goo et al., 2016).

## Modes of Transmission

### Vectorial Transmission

ZIKV was isolated for the first time from a sentinel allocthonous monkey in Uganda in 1948; just 1 year later the sylvatic *Aedes africanus* was found infected in the same site of the Zika Forest, and again in 1958, 1964, and 1969, caught both from the upper canopy and from ground level. Since then, during the period when the records are still mainly restricted to Africa many *Aedes* species reported harboring ZIKV: *Ae. vitatus, Ae. hirsutus, Ae. unilineatus, Ae. metallicus. Ae. apicoergenteus, Ae. opok, Ae. dalzieli, Ae. luteocephalus, Ae. tayliri* (Haddow et al., 1964; Hayes, 2009; Faye et al., 2013; Diallo et al., 2014; Vorou, 2016). Out of Africa, *Ae. hensillii* was incriminated in the Yap Island as the prevalent species during the outbreak and it was demonstrated experimentally capable of transmission, while in French Polynesia *Ae. polynesium* probably contributes to the transmission (Duffy et al., 2009; Ledermann et al., 2014; Richard et al., 2016).

The scenario of potential worldwide spread of ZIKV changed radically when *Ae. aegypti* and *Ae. albopictus* were identified as vectors during the outbreak in Brazil (Ferreira-de-Brito et al., 2016). Actually, *Ae agypti* had already been demonstrated competent for ZIKV in 1956 and isolated from Malaysia in 1969. In addition, it was incriminated in the French Polynesia and Indonesia outbreaks and retrospectively in Africa (Marchette et al., 1969; Richard et al., 2016). *Ae. albopictus* was associated with the outbreak in Gabon in 2007 as this Asian mosquito successfully replace the native *Ae. aegypti* in many areas of Africa (Grard et al., 2014), and it was also shown to be a potential vector in Singapore (Wong et al., 2013). These ubiquitous two mosquito species, with their dynamic adaptation to urban environments, capacity to breed in cryptic containers, to survive to adverse seasons or to be dispersed passively by humans (adults, desiccated eggs-*Ae aegypti*), to tolerate temperate climates and even keep sylvatic niches (*Ae. albopictus*), together with the increase in the last decades of social trends as those of urbanization, traveling (speed and number of people involved), migration and climate extraordinary events, generates the current epidemic risk momentum.

Further, species that belong to genera other than *Aedes* as *Culex perfuscus, Anopheles coustani, An. gambiae s.l., Mansonia uniformis* were found infected with ZIKV in Africa (Vorou, 2016), but the isolations only prove that these mosquitoes recently fed on a viremic vertebrate. On the other hand, the vector competence is the innate capability to acquire and sustain the pathogen, and transmit it to a host, so many experiments on competence were performed in alternative vectors, mainly with *Cx pipiens* and *Cx quinquefasciatus*. The results showed arguments that support (Franca et al., 2016; Guedes et al., 2016; Guo et al., 2016) or reject (Aliota et al., 2016b; Amraoui et al., 2016; Boccolini et al., 2016; Fernandes et al., 2016; Hall-Mendelin et al., 2016; Huang Y.J. et al., 2016; Weger-Lucarelli et al., 2016; Hart et al., 2017) *Culex* vector competence. To understand so many disparate results, the protocols should be analyzed and compared taking into account several issues that include the virus-vector origin (geographical coherence), and virus and vector past history in the laboratory (number of passages/generations) that made them genetically quite different to the complex wild circulating ones (Berthet et al., 2014; Bennett et al., 2016; Chouin-Carneiro et al., 2016; Wang L. et al., 2016).

However, even if the vector competence is assessed, it is rather different from vector capacity. The last concept attempts to explain the likelihood of effective human-vector contact and transmission, and depends not only on innate vector characteristics but also on local density, host range, and blood feeding behavior, biting rate, survival rate and colonization success (interspecific competence), as well as transovarial (Thangamani et al., 2016) and horizontal transmission. Therefore, even the presence of *Ae aegypti* does not confirm this species as the primary vector (Diagne et al., 2015; Di Luca et al., 2016; Weger-Lucarelli et al., 2016), and an undescribed sylvatic cycle in Asia should not be discarded (Althouse et al., 2016).

Focused in the main known urban vectors, *Ae. aegypti* and *Ae albopictus*, databases and maps of current or forecasted distribution were developed as a proxy of risk maps for ZIKV or *Aedes*-borne arbovirus (Kraemer et al., 2015a,b; Messina et al., 2016). The modeling of simulated risk is usually driven by temperature, precipitation, elevation, land cover and modulated by variables as seasonal or year-round abundance and density (population dynamics), vector biting and mortality rates, and extrinsic incubation period (Carlson et al., 2016; Escobar et al., 2016; Messina et al., 2016; Samy et al., 2016; Attaway et al., 2017). However, besides the accuracy and the assumptions of the model itself, some methodological matters require usually in-depth considerations from the mapping to assess the actual risk, as the space and time scale consistency between data and conclusions, accuracy and representativeness of field-collected data, and the particularities at smaller time or spatial scales (Jian et al., 2016; Misslin et al., 2016; Fischer et al., 2017; Li X. et al., 2017). Hence, the Latin America outbreak was explained by 2015–2016 ‘El Niño-Oscillation South’ 2015–2015 at continental level, but also at sub-regional level in Brazil it was explained by year to year variability (drought 2013–2015) and decadal variability followed by long-term trends as climate change (warm 2014–2015) (Munoz et al., 2016; Caminade et al., 2017). Furthermore, biological topics, as vector competence of local vectors (Gardner et al., 2016), vector competence between species (Camara et al., 2016), and the timing and location of vector or virus introduction (Robert et al., 2016; Walther et al., 2017) can change the probability and magnitude of transmission. Nevertheless, the anthropogenic factors usually are the main ones that trigger actual epidemics, even through climatic extreme events (Ahmed and Memish, 2017), and so some modeling in border areas includes also travel between borders and socioeconomic factors (Monaghan et al., 2016), while drivers of non-vectorial transmission still need better epidemiological elucidation (Guzzetta et al., 2016).

### Non-vectorial Transmission

Since the first report of probable sexual transmission of ZIKV by Foy et al. (2011), many studies were published showing evidence of male-to-female, male-to-male and female-to-male sexual transmission by unprotected vaginal, oral or anal intercourse (Moreira et al., 2017). This hypothesis has been strengthened by numerous reports showing the long-term detection of ZIKV RNA and the isolation of infectious ZIKV from semen (Musso et al., 2015; Matheron et al., 2016; Nicastri et al., 2016; Moreira et al., 2017). ZIKV RNA has also been detected in female genital tract samples beyond viremia, albeit more transiently than in semen: it became undetectable around 3 weeks after symptom onset (Prisant et al., 2016, 2017; Murray et al., 2017). Moreover, Penot et al. (2017) have isolated infectious ZIKV from vaginal samples collected 3 days after the onset of symptoms from a woman with controlled HIV infection.

Remarkably, the vertical transmission of ZIKV (Besnard et al., 2014; Brasil et al., 2016; Calvet et al., 2016; Driggers et al., 2016; Oliveira Melo et al., 2016) has become a major public health challenge, as will be discussed below. It still needs to be demonstrated, however, whether ZIKV can be transmitted through breastfeeding. Infectious ZIKV particles have been isolated from the breast milk of 1 mother and ZIKV RNA was detected in the breast milk of 3 symptomatic mothers (Besnard et al., 2014; Dupont-Rouzeyrol et al., 2016). Other reported forms of non-vectorial transmission include non-sexual person-to-person contact (Swaminathan et al., 2016), and transmission by blood transfusion (Motta et al., 2016).

## Clinical Manifestations

**FIGURE 4:**
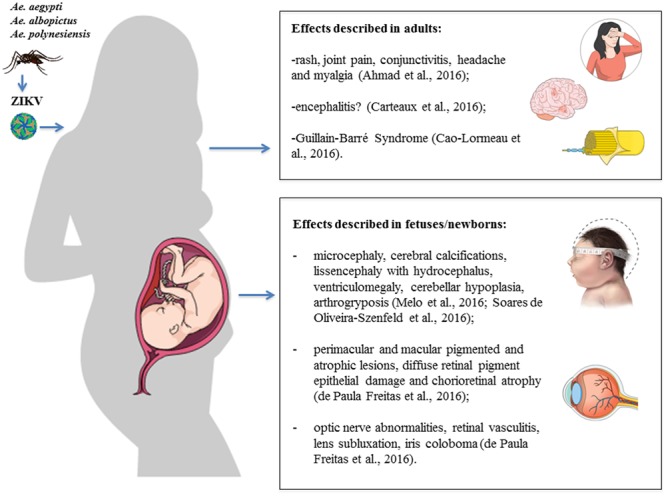
Clinical manifestations and consequences of Zika virus (ZIKV) infection. Schematic illustration of the effects described in adults and fetuses/newborns after ZIKV infection. Figure created in the Mind the Graph platform.

It has been estimated that the ZIKV infection may be symptomatic in 18–57% of cases, in which it causes a mild, self-limiting disease with an incubation period of up to 10 days (Duffy et al., 2009; Aubry et al., 2017). Symptomatic patients may develop fever and influenza-like symptoms relatively common in arboviral infections, such as rash, joint pain, conjunctivitis, headache and myalgia (Ahmad et al., 2016). These relatively mild symptoms last a few days and uncommonly result in hospitalization (Duffy et al., 2009). More recently, however, ZIKV infection has been associated with neurological and ophthalmological complications, including GBS in adults and microcephaly in fetus and newborns (**Figure 4**).

### Neurological Complications in Adults

Two cases of encephalopathy syndromes, with seizures or electroencephalographic changes, were seen in Martinique as part of ZIKV infection, probably due to encephalitis (Roze et al., 2016). Carteaux et al. (2016) reported a case of an 81-year old man from France who was admitted to an Intensive Care Unit 10 days after a cruise to Pacific islands, with fever and decreased level of consciousness, being diagnosed with meningoencephalitis and had a positive RT-PCR for ZIKV in his cerebrospinal fluid. The patient later recovered and was discharged after 17 days, with cognitive function fully recovered after 38 days and residual weakness in his left arm. In Brazil, a 47-year old pregnant patient was admitted to an Intensive Care Unit with confusion, dysarthria and lower limb weakness, 4 days after presenting a rash and arthralgia (Soares et al., 2016). The patient was diagnosed with encephalitis and had a positive PCR for ZIKV in her urine and IgM ZIKV antibody in her cerebrospinal fluid and serum, and passed away after 11 days (Soares et al., 2016). Since these observations have only recently been described, very little is known about the frequency of a direct central nervous system (CNS) infection by ZIKV. A possible confounder is the simultaneous occurrence of various arboviruses, such as Chikungunya and DENV, since these arboviruses can cause a direct CNS invasion with myelitis, encephalitis, and meningitis (Moulin et al., 2016).

Throughout the ZIKV epidemic in French Polynesia, an increase in the number of patients presenting with GBS was seen (Paploski et al., 2016). Other arboviral infections such as dengue, chikungunya, Japanese encephalitis, and West Nile fever (WNF) have been associated with GBS (Ravi et al., 1994; Lebrun et al., 2009; Leis and Stokic, 2012; Verma et al., 2014). Cao-Lormeau et al. (2016) published a case-control study reporting the occurrence of 42 cases of GBS after a Zika outbreak between October 2013 and April 2014, in comparison to 5–10 cases in the same period in previous years. All 42 patients had neutralizing antibodies against ZIKV, whereas only 56% of neutralizing antibodies were found in the serum from a control group of 98 patients without GBS. The majority of GBS patients (93%) had detectable ZIKV IgM and 88% had a systemic febrile disease with symptoms that corresponded to ZIKV infection before the development of the neurological symptoms. This cohort of ZIKV associated GBS was classified electrophysiologically as acute motor axonal neuropathy (AMAN) with a rapid onset of disease (4 days to reach the plateau). Approximately one-third of patients required intensive medical therapy with mechanical ventilation (Cao-Lormeau et al., 2016). Anti-glycolipid antibodies were found in 31% of patients. However, anti-ganglioside antibodies typical of AMAN were rarely present (Cao-Lormeau et al., 2016). Dos Santos et al. (2016) performed an analysis of ministry of health websites and International Health Regulations channels to compare the occurrence of GBS before and after the Zika outbreak. They found an increase of 877% in Venezuela, 400% in Suriname, 211% in Colombia, 172% in the Brazilian state of Bahia, 150 % in the Dominican Republic, 144% in Honduras and 100% in El Salvador.

### Neurological Complications in Newborns

Zika infection has been confirmed in newborns with microcephaly, and an up to 20-fold increase in the number of microcephaly cases in the French Polynesia, Brazil and other Latin American countries (Brasil et al., 2016; Jouannic et al., 2016; Schuler-Faccini et al., 2016; Ventura et al., 2016a). After the French Polynesia epidemic between October 2013 and April 2014, 14 cases of fetuses and newborns with brain abnormalities (six of them without microcephaly) and five cases with brainstem dysfunction were observed, much higher numbers than those expected outside the epidemic period. Symptomatic cases were related to infections acquired during the first trimester, which is consistent with other congenital infections, such as rubella, cytomegalovirus, and toxoplasmosis (Besnard et al., 2016). However, some severe forms have been described in infections acquired after 20 weeks of gestation (Brasil et al., 2016). There is not always evidence of infection of mothers or fetuses by ZIKV but its presence in the amniotic fluid has been documented (Brasil et al., 2016). The rate of fetal transmission and the actual incidence of serious congenital infections remain unclear. The virus has been detected in fetal cerebral tissues by RT-PCR and electron microscopy (Mlakar et al., 2016).

Brasil et al. (2016) included 345 pregnant women with a history of rash within 5 previous days in a cohort study from September 2015 to May 2016. A total of 182 (53%) tested positive for ZIKV in the blood, urine, or both, and outcomes were available for 125 of these patients. There were nine fetal deaths, and the remaining 116 ZIKV-positive women gave birth to 117 live infants, 42% of which had grossly altered clinical and/or neuroimaging findings, four of them with microcephaly. Melo et al. (2016) followed 11 infants with congenital ZIKV syndrome from gestation to 6 months in Brazil, identifying neurological injuries that included lissencephaly with hydrocephalus, ventriculomegaly, microcephaly, reduction in cerebral volume, cerebellar hypoplasia and arthrogryposis. Soares de Oliveira-Szejnfeld et al. (2016) performed radiological investigation in 17 patients with confirmed ZIKV infection and 18 with presumed ZIKV. The authors reported that neuroimaging abnormalities were similar between both groups, with the more frequent being ventriculomegaly, corpus callosum, infections, intracranial calcifications in the junction of the gray-white matter or in the thalamus and/or basal ganglia.

### Ophthalmological Complications

Ventura et al. (2016a) first described ocular lesions in three infants of ZIKV-infected mothers in Brazil, who presented with microcephaly and cerebral calcifications. Mothers and their children underwent ophthalmological evaluation by biomicroscopy and fundus examination. None of the infected mothers presented eye lesions; however, their infants had gross pigment mottle in the macula and no foveal reflex. ZIKV infection was not tested by RT-PCR, but this was the first warning of its deleterious effects on the developing visual system. The same group also reported on a 57-day infant without microcephaly that had chorioretinal scar on the macula of the left eye (Ventura et al., 2016b). Later, the same group published a study with 40 infants with microcephaly, and reported that eye lesions were more frequent in infants whose mothers had symptoms during the first trimester and infants who had smaller cephalic diameters (Ventura et al., 2016c).

A report by de Paula Freitas et al. (2016) evaluated 23 out of 27 women with suspected infection by ZIKV who had presented clinical manifestations during gestation. Eighteen of them presented them during the first trimester; four occurred in the second trimester and one in the third trimester of gestation. The newborns were studied by ophthalmic examination and, of 29 children (58 eyes), 10 (35.5%) ocular abnormalities were identified in 17 eyes (29.3%). The main fundus results were perimacular and macular pigmented and atrophic lesions, diffuse retinal pigment epithelial damage and chorioretinal atrophy, which in some cases presented very severe forms affecting the macula. They also reported the occurrence of optic nerve abnormalities, mainly hypoplasia, and other findings such as retinal vasculitis, lens subluxation and iris coloboma. In the study, the authors described bilateral findings in 2/3 of the children examined. The authors determined that congenital ZIKV infection was associated with potentially blinding eye disease, including bilateral irreversible macular and perimacular lesions and involvement of the optic nerve (de Paula Freitas et al., 2016).

## Models of ZIKV-Induced Neural Damage

Well before the first suspected cases of ZIKV-associated neurological disorders in humans, there were reports showing the marked neurotropism of ZIKV strains isolated in Uganda (Dick, 1952; Bell et al., 1971). These studies briefly described the neuropathological changes induced by the intracerebral inoculation of ZIKV in newborn and adult mice, such as the severe neuronal degeneration and reactive astrocytosis in the hippocampus of newborn Webster Swiss mice inoculated with ZIKV (Bell et al., 1971). Only recently, however, after the recent outbreak of ZIKV in South and Central America, the study of the vulnerability of neural cells to ZIKV infection has become a focus of intense research.

### *In Vitro* and *Ex Vivo* Models of ZIKV Infection in Neural Cells and Tissues

*In vitro* studies using human induced pluripotent stem cells (iPSC) have allowed the investigation of the consequences of ZIKV infection in different types of human neural stem cells (NSC), neural progenitor cells (NPC) and their progeny, as well as in cerebral organoids (Cugola et al., 2016; Garcez et al., 2016; Souza et al., 2016; Tang et al., 2016). Human organotypic fetal brain slices (Onorati et al., 2016; Retallack et al., 2016), human NPC derived from the fetal brain (Hanners et al., 2016; Liang et al., 2016; Onorati et al., 2016) and immortalized cells lines also have been employed to study the neurovirulence of ZIKV.

Tang et al. (2016) were the first to show that the MR766 strain of ZIKV (from Uganda) infected iPSC-derived forebrain-specific human NPC, leading to cell-cycle dysregulation and apoptosis, whereas human iPSC and immature neurons exhibited lower levels of infection. In line with these evidence, Garcez et al. (2016) showed that, while both ZIKV (MR766 strain) and DENV 2 (16681 strain) were capable of infecting human NSC, only ZIKV induced apoptosis in NSC, impaired the formation of neurospheres and decreased the growth rate of human brain organoids. Further studies revealed that brain organoids represent an interesting platform for the study of ZIKV-associated microcephaly (Cugola et al., 2016; Dang et al., 2016; Qian et al., 2016; Wells et al., 2016; Gabriel et al., 2017). For instance, Qian et al. (2016) exposed brain organoids at different stages of cortical neurogenesis to ZIKV (MR766) for 24 h. They showed that most of the infected cells were NPC, although the virus could be detected to a lesser extent in immature neurons, intermediate progenitor cells, and astrocytes. Infection of early stage organoids decreased the number of proliferating cells and induced apoptosis in infected and non-infected cells. As a consequence, the ventricular zone and the neuronal layer were thinner and the ventricles were enlarged in infected organoids, resembling some of the characteristics of microcephaly.

#### ZIKV Induces Apoptosis, Autophagy and Mitotic Abnormalities in NSC/NPC

Studies using an Asian strain of ZIKV (FSS13025, isolated in Cambodia) (Zhang F. et al., 2016) or ZIKV isolated from recent outbreaks in Brazil (Cugola et al., 2016; Souza et al., 2016; Garcez et al., 2017; Sacramento et al., 2017), Puerto Rico (Hanners et al., 2016; Wells et al., 2016) and French Polynesia (Ghouzzi et al., 2016) have shown the ability of the virus to infect and induce apoptosis of human NPC.

Another common feature of the different strains of ZIKV is the capacity to inhibit the proliferation of NPC. In this regard, Liang et al. (2016) screened the effects of ten ZIKV-encoding potential proteins and found that the ectopic expression of two proteins (NS4A and NS4B, alone or in combination) inhibited neurosphere formation, decreased the proliferation rates of NSC derived from human fetuses, and reduced their capacity to differentiate into neurons and astrocytes. They showed that the co-expression of NS4A and NS4B induced autophagy, by impairing Akt/mTOR signaling, and suggested that the efficient replication of ZIKV requires autophagy (Liang et al., 2016). Infection with either the Brazilian ZIKV or the Cambodian ZIKV strain FSS 13025 caused mitotic abnormalities and increased the number of neural stem/progenitor cells with supernumerary centrosomes (Onorati et al., 2016; Souza et al., 2016; Garcez et al., 2017). Supernumerary foci of centriolar proteins have also been found after ZIKV infection (Polynesia strain PF-25013-18) in untransformed human retinal epithelia RPE-1 cells and human CHME3 microglial cells, but not in the NPC line ReN (Wolf et al., 2017). Onorati et al. (2016) have proposed a model in which ZIKV infection activates RIG-I-like receptors, cytoplasmic sensors of different forms of dsRNA (which are present during the replication of ssRNA virus) (Thompson and Locarnini, 2007). This would, in turn, cause the relocation of phosphorylated TANK-binding kinase 1 (pTBK1) from centrosomes to mitochondria - where pTBK1 could take part in an innate antiviral immune response – resulting in mitotic impairments in neocortical neuroepithelial stem cells and radial glial cells (Onorati et al., 2016). Interestingly, the relocation of pTBK1 to the mitochondria was also induced by human cytomegalovirus (HCMV), another TORCH syndrome pathogen, but not by DENV 2 (16681 strain), despite the fact that both viruses induced apoptosis in human neocortical neuroepithelial stem cells derived from prenatal specimens (Onorati et al., 2016). The importance of ZIKV-induced centrosomal abnormalities was reinforced by recent findings from Gabriel et al. (Gabriel et al., 2017), who suggested a link between centrosomal structural defects in ZIKV-infected human NPC and the premature differentiation of NPC, which would result in the depletion of the NPC pool.

#### Mechanisms of ZIKV Entry in Human Cells and the Antiviral Response

Although the knowledge of the cell biology of ZIKV is still scarce, recent advances have provided insights on the life cycle of this pathogen. ZIKV can bind to target cells using adhesion factors such as DC-SIGN and phosphatidylserine binding receptors (Hamel et al., 2017) from which Axl appeared as the main receptor for the entry in human skin fibroblasts (Hamel et al., 2015), microglia (Meertens et al., 2017), astrocytes (Retallack et al., 2016; Meertens et al., 2017) and blood-brain barrier endothelial cells (Liu et al., 2016). Surprisingly, Axl does not seem to be necessary for the entry of ZIKV in NPC and cerebral organoids (Wells et al., 2016; Meertens et al., 2017), despite the high expression of this receptor in NSC and NPC (Cugola et al., 2016; Liu et al., 2016; Nowakowski et al., 2016; Onorati et al., 2016; Meertens et al., 2017). Replication and assembly of progeny virions of ZIKV take place onto modified membranes derived from the endoplasmic reticulum. Thus, ZIKV-infected cells exhibit the characteristic ultrastructural alterations of flavivirus-infected cells (Dick, 1952; Offerdahl et al., 2017). In addition, ZIKV infection provokes changes in the pattern of cellular and viral RNA methylation (Lichinchi et al., 2016) and induces a major impact on the transcriptome of the host cell (Rolfe et al., 2016).

It has been shown that ZIKV (MR766) infection increases the expression of Toll-like receptor 3 (TLR3) (Dang et al., 2016), another innate immune receptor that recognizes dsRNA (Thompson and Locarnini, 2007), in iPS-derived human cerebral organoids and neurospheres. Treatment with the TLR3 agonist poly(I:C) decreased the size of neurospheres in a similar fashion to the infection with ZIKV. Nevertheless, treatment with a TLR3 competitive inhibitor only provided a modest protection against the deleterious effects of ZIKV in neurospheres and cerebral organoids (Dang et al., 2016). In another study, it was shown that while poly(I:C) induced the secretion of inflammatory mediators by human NPC, infection with a ZIKV strain isolated in Puerto Rico (ZIKV-PRVABC59) was not capable of inducing such a response – despite inducing apoptosis. In addition, ZIKV did not induce a type I Interferon response in NPC (did not induce IFN-α secretion) and did not stimulate cytokine secretion in THP-1 human monocytic cells (Hanners et al., 2016). ZIKV infection (MR766) was also shown to downregulate several immune response genes in a human microglial cell line (Tiwari et al., 2017). Suppression of the innate immune response in human CHME3 microglial cells infected with ZIKV was shown to depend on the kinase activity of Axl (Meertens et al., 2017).

Other studies, however, have found that ZIKV is capable of inducing an immune response in human fetal brain microglia (Lum et al., 2017) and other cell types. Human embryonic stem cells-derived cranial neural crest cells (CNCC) were induced to secrete high concentrations of several cytokines and growth factors, such as IL-6, PAI-1, LIF, and VEGF, after the infection with ZIKV (MR766 or H/PF/2013 strains). CNCC supported ZIKV replication and only a small fraction of the cells died after infection (Bayless et al., 2016). Moreover, ZIKV (PF-25013-18) induced the upregulation of the transcription factor IRF7 and several interferon-stimulated genes in human skin fibroblasts and lung epithelial A549 cells (Hamel et al., 2015; Frumence et al., 2016). Interestingly, the pattern of antiviral response induction in human astrocytes can differ between two ZIKV strains (H/PF/2013 and African HD 78788 strains) (Hamel et al., 2017).

#### Are There any Differences in the Neurovirulence of Different ZIKV Strains?

**FIGURE 5:**
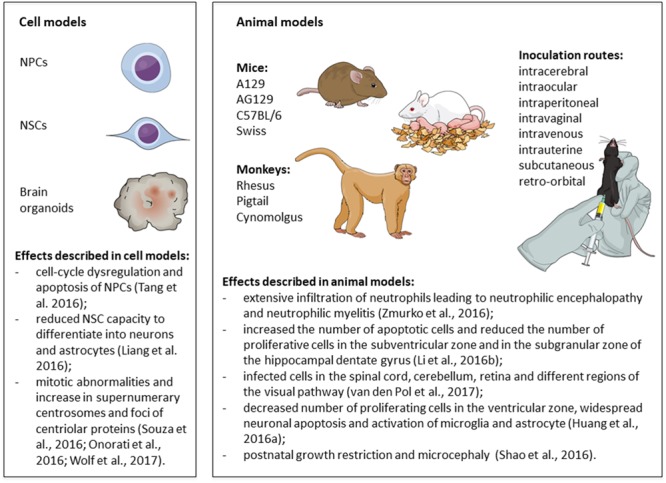
Mechanisms of Zika virus (ZIKV)-induced neural damage. Schematic illustration of the main findings from *in vitro* and *in vivo* models of Zika virus infection. Figure created in the Mind the Graph platform. NPCs: neural progenitor cells; NSCs: neural stem cells.

Taken together, current evidence indicates that ZIKV preferentially infects NPC, inducing apoptosis, autophagy and interfering with mitosis (**Figure 5**). ZIKV replicates in NPC and the surviving cells produce the virus for several weeks (Hanners et al., 2016). All strains of ZIKV currently tested have been shown to induce nearly the same cytopathological effects in NPC and few studies have addressed whether there are mechanistic differences among the strains. Cugola et al. (2016) showed that, while the African strain reduced the number of neurons in non-human primate cerebral organoids, the Brazilian strain failed to replicate and did not change the number of neurons, suggesting that the Brazilian virus strain underwent adaptive changes in human cells. Gabriel et al. (2017) have also found some differences in the cellular outcome by comparing the effects of two strains of ZIKV isolated during the recent outbreaks and the African strain MR766 in iPSC-derived human NPC and cerebral organoids. Furthermore, Zhang F. et al. (2016) compared the effects of infecting iPSC-derived human NPC with an Asian ZIKV isolate (FSS13025) or the African strain (MR766). Both strains induced the same alterations in NPC (cell death and decreased proliferation) and caused similar gene expression changes: downregulation of genes involved in cell cycle, DNA repair, and DNA replication, coupled to the upregulation of genes involved in cell death and unfolded protein responses. Comparing the effect of both strains, they found that TP53 (coding for the tumor suppressor protein p53) was significantly upregulated following the infection with the Asian strain, but only marginally upregulated after the infection with the African strain. Accordingly, p53 inhibitors reduced the activation of caspase-3 more efficiently in NPC infected with the Asian strain (Zhang F. et al., 2016). Activation of p53 has also been reported after the infection of iPSC-derived human NPC with a ZIKV strain isolated from French Polynesia (PF13). The activation of p53 was not restricted to NPC expressing high levels of viral antigens (Ghouzzi et al., 2016), suggesting that this might be an early event or an indirect effect of ZIKV infection in neighboring cells. Other cell types, such as microglia, astrocytes, and endothelial cells, can also be infected (Delvecchio et al., 2016; Lum et al., 2017; Meertens et al., 2017), but it is unknown how the interplay among different cell types contribute to the outcome of microcephaly.

### Animal Models of ZIKV Infection

#### Immunodeficient Mouse Models

Several studies have used mice deficient in type I (A129 mice) or type I/II interferon receptors (AG129) to establish models of robust ZIKV infection in juvenile and adult animals. Aliota et al. (2016a) showed that the subcutaneous injection of a French Polynesian strain of ZIKV produced a lethal infection in AG129 mice. Animals exhibited early serum viremia and high viral loads in several organs, which was accompanied by signs of illness (including weight loss and lethargy) and brain degeneration. One of the most striking observations was the extensive infiltration of neutrophils in the brain. Animals had to be euthanized 7–8 days post-infection due to the rapid progression of the disease. Zmurko et al. (2016) infected AG129 mice with the African strain of ZIKV (MR766) and reported that the first signs of disease appeared at 10 days after the intraperitoneal inoculation. Mice developed acute neutrophilic encephalopathy and had to be euthanized, on average, at 14 days post-inoculation. Acute multifocal neutrophilic encephalitis, inflammatory lesions in the cerebellum and multifocal neutrophilic myelitis were also observed after the subcutaneous inoculation of AG129 mice with the Malaysian strain of ZIKV (P 6-740). In this model, all mice died within 21 days after the infection (Julander et al., 2017). Similar findings were reported after the infection of A129 mice with an African strain (MP1751) (Dowall et al., 2016), a Cambodian strain (FSS13025) (Rossi et al., 2016), or a French Polynesian strain of ZIKV (H/PF/2013) (Lazear et al., 2016). Rossi et al. (2016) compared the effects of ZIKV infection in both mouse strains and found that AG129 mice had more severe neurological symptoms than A129 mice, although there were no differences in other parameters, such as time to death and weight change, suggesting that type II interferon signaling might also influence certain aspects of the disease. Lazear et al. (2016) established a lethal infection in triple knockout mice deficient for IRF3, IRF5 and IRF7, which virtually do not produce interferon-α/β, and showed that these mice are more vulnerable to the intravenous injection of ZIKV than A129 mice, although there were no differences when the subcutaneous route was used. They also showed that the African strain MR766 was less pathogenic to A129 mice than the French Polynesian ZIKV strain (H/PF/2013). Another study using triple knockout mice (IRF3, IRF5, and IRF7 knockout) observed that ZIKV also has a tropism for NPC and immature neurons in the adult brain (Li H. et al., 2016). In this study, mice infected with a Cambodian strain of ZIKV (FSS13025) through a retro-orbital injection exhibited signs of viral illness and hindlimb weakness. The viral envelope protein was detected mainly in the subventricular zone and in the subgranular zone of the hippocampal dentate gyrus, two neurogenic regions in adults. Immunohistochemical analysis revealed that the infection increased the number of apoptotic cells and reduced the number of proliferative cells in these two neurogenic niches.

#### Immunocompetent Mouse Models

Several studies have demonstrated that immunocompetent mouse strains (CD1, C57BL/6 and 129Sv/Ev mice) are resistant to the subcutaneous or intraperitoneal inoculation of ZIKV and do not develop signs of illness (Dowall et al., 2016; Lazear et al., 2016; Rossi et al., 2016).

Immunocompetent mice only developed the disease when infected during the neonatal period (Lazear et al., 2016; Fernandes et al., 2017). For instance, van den Pol et al. (2017) inoculated C57BL/6 mice on the day of birth with an Asian strain of ZIKV (FSS13025) via the intraperitoneal route. They found that astrocytes were the first targets of the virus in the brain, which was followed by the infection of neurons. Infected cells were also found in the spinal cord, cerebellum, retina and different regions of the visual pathway. Infection was often lethal and caused growth restriction and motor dysfunction.

Fernandes et al. (2017) inoculated newborn Swiss mice with ZIKV (Brazilian strain SPH 2015), through the intracerebral or the subcutaneous routes. Animals from both groups showed neurological symptoms and severe illness, but the disease progressed faster in the intracerebral group. Histopathological findings were similar to what had been described in A129 and AG129 juvenile and adult animals. An interesting observation was that animals infected through the subcutaneous route additionally exhibited myelopathy, although the brain injury was less severe. Huang W. C. et al. (2016) infected C57BL/6 mice with ZIKV (MR766) at two different time points (either at postnatal day 7 or 21) through the intracerebral route. Infection of postnatal day 7 mice decreased the number of proliferating cells in the ventricular zone and resulted in widespread neuronal apoptosis and in the activation of microglia and astrocytes throughout the brain at 4 days post-infection, when the animals already exhibited neurological symptoms. At this time point, 21-day-old infected mice had severe paralysis, but the regional pattern of neuronal apoptosis was different and less prominent.

Three studies have also reported the effects of injecting ZIKV into the lateral ventricles of C57BL/6 and 129S1/SvImJ mouse embryos. Shao et al. (2016) observed postnatal growth restriction and microcephaly in newborn pups infected with a Mexican strain of ZIKV (MEX1-44) at embryonic day 14.5. Microscopic analysis of their brains revealed cortical thinning, massive neuronal death, glial activation and abnormal vascular density and permeability. Li C. et al. (2016) injected an Asian strain of ZIKV (SZ01) in the lateral ventricles of embryonic day 13.5 mice. They confirmed the tropism of ZIKV for NPC, although at a later time-point (5 days post-infection) almost all cells in the brain were positive for ZIKV. They also found that ZIKV induced the death of immature and mature neurons and reduced NPC proliferation and differentiation, which resulted in a thinner cortical layer. In both studies, there was no evidence of disruption of cortical lamination and the global transcriptome analyses of infected brains showed the upregulation of genes involved in immune-response-related and apoptosis pathways (Li C. et al., 2016; Shao et al., 2016). Wu et al. (2016), however, found the virus mainly in the ventricular zone and striatum of postnatal day 1 mice that were infected at embryonic day 13.5.

Thus far, animal models corroborate the evidence from *in vitro* and *ex vivo* studies, indicating that NPC are highly susceptible to ZIKV infection (**Figure 5**). Postmitotic neurons were also shown to be targets of infection in the developing brain and spinal cord of immunocompetent animals and in the brain of young and adult transgenic mice that do not mount an antiviral interferon response. Hindlimb paralysis and neurological symptoms were found in almost all models. Moreover, these models allowed the investigation of important questions raised by clinical and epidemiological observations, such as the link between ZIKV infection during pregnancy and the occurrence of microcephaly, and the possibility of sexual transmission of ZIKV.

#### Models of Sexual and Vertical Transmissions

The vertical transmission of ZIKV requires the ability of the virus to cross the placental barrier, as well as the developing or developed blood-brain barrier (BBB). Cugola et al. (2016) infected C57BL/6 or SJL pregnant mice on day 10–13 of gestation with a Brazilian strain of ZIKV. Newborns from the SJL ZIKV-infected mice had a high viral load in the brain, intra-uterine growth restriction (IUGR), cortical malformations and ocular defects. These alterations, however, were not observed in pups from C57BL/6 ZIKV-infected mice, indicating that the virus was not able to cross the placenta in this mouse strain, a finding that was supported by a recent study by van den Pol et al. (2017). This is in contrast with the findings of Wu et al. (2016), who injected ZIKV isolated from a patient contaminated in Samoa in the peritoneal cavity of pregnant C57BL/6 mice at embryonic day 13.5. They detected viral RNA in 5 out of 9 placentas and found the virus in radial glial cells localized in the ventricular zone of the fetal brains, 3 days after infection. ZIKV infection reduced the number of proliferating cells in the ventricular zone and intermediate zone and decreased the outer perimeter of the cortex of mice fetuses, although there was no difference in relative thickness of cortical layers. Recently, Xavier-Neto et al. (2017) showed a window of susceptibility between 5.5 and 9.5 days *post coitum* for ZIKV-induced teratogenesis in FVB/NJ and C57BL/6J WT mice. Their model of ZIKV (HS-2015-BA-01, isolated from a Brazilian patient) injection through the jugular vein of pregnant mice caused gross and generalized malformations, IUGR and neural tube defects in the embryos/fetuses.

Another strategy was employed by Miner et al. (2016) who crossed A129 female mice with wild-type (WT) males. Pregnant mice were infected with ZIKV (H/PF/2013) through the subcutaneous route on embryonic days 6.5 or 7.5. This model resulted in high levels of ZIKV RNA in the placenta (1000-fold greater than in the blood), placental abnormalities (vascular injury and apoptosis of trophoblasts) and fetal demise and resorption. IUGR and a large number of apoptotic cells in the brain were found in the remaining fetuses. Alternatively, they treated pregnant mice with a blocking anti-interferon alpha/beta receptor subunit 1 (MAR1-5A3) 24 or 48 h before the inoculation o ZIKV at embryonic days 6.5 or 7.5. This protocol did not cause fetal demise but caused mild IUGR and placental and fetal infection.

Another interesting observation from animal studies is that the brain and testes are the main sites of ZIKV replication in juvenile A129 and AG129 mice (Rossi et al., 2016) and that the virus can persist in the brain and testes for up to 28 days after infection in adult A129 mice (Lazear et al., 2016). Importantly, ZIKV infection (African strain Dakar 41519, and Asian strain H/PF/2013), although at lower levels, caused testis damage, oligospermia and decreased sex hormone production in WT mice treated with anti-Ifnar1 blocking antibodies (Govero et al., 2016). Testicular damage was also observed after the intraperitoneal inoculation of ZIKV (Asian strain SMGC-1) in A129 mice and after the intratesticular injection of ZIKV in WT mice (Ma et al., 2016). Future studies are warranted to determine whether the testicles are affected in ZIKV-infected men.

The possibility of ZIKV transmission through sexual contact was investigated by Yockey et al. (2016), who demonstrated that ZIKV (Cambodian strain FSS13025) replicates in the vaginal tract of WT mice after the intravaginal inoculation. However, while WT mice did not develop disease, A129 mice developed hindlimb paralysis and died within 9 days. However, it was shown that this model of intravaginal infection in WT mice can lead to IUGR if the maternal infection occurs during early pregnancy, even in the absence of viremia. The presence of ZIKV in neurons and glial cells in the fetal brain was also demonstrated. On the other hand, vaginal ZIKV exposure of pregnant A129 mice resulted in viremia, placental infection and fetal demise (early infection at embryonic day 4.5) or severe IUGR (infection at embryonic day 8.5). In conclusion, it has been shown that ZIKV can infect the fetal brain and cause developmental abnormalities (IUGR, for instance) in immunocompetent fetuses if there is a high viral load in the maternal vagina and/or blood, or if the virus is directly inoculated in the brain. Finally, Vermillion et al. (2017) developed a model of transplacental transmission in immunocompetent CD1 mice, in which ZIKV was directly inoculated into the uterine wall at embryonic day 10. At postnatal day 0, they found evidence of cortical thinning and microglial activation in the brain of pups from ZIKV-infected dams.

#### Non-human Primate Models and Alternative Models

Although the models that use animals that do not mount an interferon response might not be useful for the screening of compounds that target the components of this anti-viral pathway, these models might be useful for testing other therapeutic strategies. Indeed, there is evidence that the ZIKV NS5 protein inhibits type I interferon response in human cells in a species-specific fashion, which might explain why WT mice are more resistant to the infection (Grant et al., 2016). Studies using animal models, therefore, represent an important step for the development of new therapies, especially when in conjunction with *in vitro* and *ex vivo* models (Delvecchio et al., 2016; Retallack et al., 2016; Zmurko et al., 2016; Julander et al., 2017; Sacramento et al., 2017). This includes the utilization of non-human primate models in preclinical translational studies. It has been shown that the infection of Indian-origin rhesus macaques with French Polynesian, South American, Puerto Rican or Thai ZIKV strains, via the subcutaneous route, results in transient viremia and uremia, prolonged presence of ZIKV RNA in cerebrospinal fluid, lymph nodes and colorectal tissue, as well as in the persistent presence of infectious ZIKV in the semen (Dudley et al., 2016; Li X.F. et al., 2016; Osuna et al., 2016; Aid et al., 2017). ZIKV infection induced T-cell responses and protected non-human primates from ZIKV re-infection or from heterologous ZIKV infection (Dudley et al., 2016; Osuna et al., 2016). Pregnant macaques infected at mid-first semester, however, exhibited persistent viremia, although the amniotic fluid was negative for ZIKV (Dudley et al., 2016). Moreover, Adams Waldorf et al. (2016) showed the vertical transmission of ZIKV (strain FSS13025, from Cambodia) after the subcutaneous inoculation of a pregnant pigtail macaque at 119 days of gestation. This model caused fetal brain lesions, including white matter injury and gliosis, brain growth arrest and an increase in the number of apoptotic cells in the subependymal zone, a neurogenic niche. ZIKV RNA was detected in both maternal and fetal brains and in the placenta at 6 weeks after inoculation, which might explain the persistent maternal viremia observed in other studies. Models of ZIKV infection in Cynomolgus monkeys have also been developed (Koide et al., 2016; Osuna et al., 2016), showing that these animals are susceptible to infection to ZIKV isolates from Cambodia and Puerto Rico, but not to an African ZIKV strain (IBH30656). In search of alternative and less expensive models, Goodfellow et al. (2016) have demonstrated that ZIKV induced a microcephaly like phenotype in chicken embryos. Finally, a model of intraocular ZIKV inoculation in mice (van den Pol et al., 2017) has already been developed and will permit the investigation of ZIKV-induced dysfunction of the retina and visual pathway.

#### ZIKV as a Causative Agent of Neurological Pathologies

Taken together, the large amount of data generated over the last years indicate that ZIKV can cause neural damage in an age-dependent manner. The causal relationship between ZIKV and birth defects has been inferred by using the Shepard’s criteria for proof of human teratogenicity and the Bradford Hill’s criteria for evidence of causation (Rasmussen et al., 2016). The publication of several experimental studies in animals showing the maternal-fetal transmission of ZIKV and the consequences of congenital infection have reinforced this conclusion. By analyzing data from epidemiologic and experimental studies, Krauer et al. (2017) concluded that ZIKV is a trigger of GBS and found evidence of causality between ZIKV and congenital abnormalities. However, the full spectrum of neurological complications and the long-term sequelae of ZIKV infection, even in the absence of microcephaly, remain to be determined. The mechanisms of neural damage at different developmental stages and the contribution of potential cofactors (nutritional status, viral load, previous infection with DENV, etc.) to the development of adverse outcomes also deserve further investigation.

## Public Health Response And Disease Control Measures

### Declaration of a PHEIC

Since the beginning of the 21st century, a number of infectious disease threats have emerged that were deemed to be such a risk that they demanded a global response. Although the transmission dynamics of the various pathogens causing these global emergencies differed, the overarching principles of the public health response were the same – development of policies and procedures, risk communication, effective surveillance, and use of disease control measures to mitigate the risk of infection in the population. These principles formed the bulk of the advice by the Emergency Committee on ZIKV to the Director-General of the WHO when the association between ZIKV and microcephaly and other neurological disorders (e.g., GBS) were declared to be a PHEIC on 1 February 2016 (WHO, 2016e). In addition to the public health measures, other key elements of that advice were that Zika-affected countries should prepare health services; research and development efforts should be increased; national authorities should ensure the rapid and timely reporting and sharing of information of public health importance relevant to this PHEIC; and there should be no restrictions on travel and trade as a result of the outbreak.

### Policies and Protocols

Because of the novel and unanticipated nature of the public health emergency relating to ZIKV, there were no specific pre-existing policies and protocols to guide the public health response. In December 2015, the European Centre for Disease Prevention and Control published a rapid risk assessment on the unfolding epidemic (ECDC, 2015) and, soon after, disease control agencies around the world developed public health and clinical guidelines. These guidelines were largely focused on women of reproductive age, their partners, and their clinicians, providing advice on how infection could be prevented, and on testing and management in the event of possible exposure (Petersen et al., 2016; WHO, 2016b). Microbiological testing for ZIKV is not straightforward and was not widely available at the outset of the epidemic, so the development of laboratory guidelines was another early focus.

Guidelines development accelerated following the WHO announcement that the world was experiencing a PHEIC. And as the scientific understanding of the risk and clinical manifestations of ZIKV infection on unborn children was rapidly evolving, guidelines required frequent updating. Over time, the guidelines for various agencies became more consistent with one another.

### Surveillance

Surveillance activities for ZIKV in areas where transmission is occurring focus on recording case numbers, complications of the infection, and vectors. The objectives of the surveillance for human cases are to monitor the geographical distribution and temporal trend of infection; characterize disease presentation; identify complications related to the infection; identify non-vector borne routes of transmission; and monitor the effectiveness of containment measures (WHO, 2016d). In countries which are receptive to the virus but without local transmission, the key surveillance objectives are to ascertain imported cases and undertake vector monitoring. In countries where there is no chance of local transmission by vectors, the focus is on identifying imported cases.

The transmission dynamics of ZIKV mean that when it is introduced to an immunologically naive population, an epidemic ensues until a level of population immunity is reached that sees the end of the outbreak. Modeling shows that this period will last approximately 3 years, with a further decade or more before large epidemics are once again possible (Ferguson et al., 2016). It is important for a country to know where on the epidemic curve it is at any point in time, so that the risk to the population is understood and communicated to them, control measures can be instituted, and planning health services undertaken. Although the mild nature of ZIKV disease means that few will come to the attention of health authorities, an understanding of trends can be observed if a robust surveillance system is in place that allows counting of the relatively small proportion of cases that do seek medical help. The more robust systems are those where notification of ZIKV infection to public health authorities is included in the list of reportable infectious diseases mandated by law. Population-based serological studies can be undertaken in Zika-affected countries to better understand the level of population immunity at a point in time.

Estimating the risk of complications from ZIKV is difficult as baseline data for benchmarking are often unavailable and neither accurate denominator data (number of mothers infected), nor numerator data (number of congenital malformations), is available in most of those countries directly affected by ZIKV. Robust surveillance systems for measuring rates of congenital anomalies do exist but tend to occur in countries where ZIKV is not endemic. One such system is the US Zika Pregnancy Registry (USZPR), which provides perhaps the most accurate estimate of risk – of 442 women with completed pregnancies and laboratory evidence of recent Zika infection, 6% of fetuses or infants overall had one or more brain abnormalities and 4% had a finding of microcephaly (Honein et al., 2017). The rate was the same for symptomatic as for asymptomatic women and by far the greatest risk was for women who were infected in their first trimester.

The complication of GBS is rare, estimated to be 2.4 cases per 10,000 people infected during the 2013–2014 outbreak in French Polynesia (Cao-Lormeau et al., 2016). Estimates of the incidence of GBS from a number of Central and South American countries affected by ZIKV range from 2.0 to 9.8-fold higher than pre-epidemic baselines, which pose a significant burden to communities and health systems (Dos Santos et al., 2016).

The aims of vector surveillance are to determine when and where competent vectors are active in a country, where vector control efforts should be focused, and the effectiveness of vector control programs. As a consequence of dengue fever epidemics, most affected countries can rely on existing vector control programs. However, such programs are resource-intensive and need to be well-planned to ensure an efficient use of resources. Trapping sites should be placed to ensure representativeness of the geographical area, and fixed in place to allow determination of changes in mosquito densities over time (WHO, 2016f). Sampling should involve counting of both larval and adult forms of known mosquito vectors to help inform the use of larvicides, adulticides or both. Ideally, synthesis of all the information should be combined with geographic information systems (GIS) data to allow prediction of disease transmission scenarios and focus risk communication to the public. Another important component is the periodic determination of insecticide sensitivity to help selection of authorized biocides used in control efforts.

### Communication to High-Risk Groups

Raising awareness of the risk of ZIKV infection to those groups at risk is of critical importance. The population groups to be targeted will vary according to whether they are in a region or country where there is ZIKV activity. In those areas where ZIKV transmission is occurring, the entire population needs to be aware of the disease and vigilant of the risk in order for them to be motivated to undertake risk mitigation measures. Apart from the general public, specific stakeholder groups in the risk communication strategy should include pregnant women, women of reproductive age and their partners, community organizations, schools, health care workers, the media, local and international organizations involved in reproductive health, and local policymakers (WHO, 2016c). Key messages should include prevention of unintended pregnancies by the use of reversible contraception methods; using insect repellents, mosquito nets and other mosquito avoidance measures; and assisting in local vector control activities, such as reducing the mosquito breeding sites on private property. This last issue is essential, because the majority of breeding sites of *Ae. albopictus* and *Ae. aegypti* in urbanized areas are usually found on private property, where the simple preventive measure of water source elimination by owners can significantly reduce the risk.

The advice of health ministries of some Zika-countries for women to defer pregnancy for considerable periods to lessen the risk to their newborns is unprecedented and controversial. Apart from the attendant population planning risks of a distorted population profile resulting from a diminished birth cohort, some of these countries have high rates of unplanned pregnancies, strict abortion laws, a lack of sexuality education programs in schools, and poor access to contraception, leading to difficulties in implementation of this policy (Ahmed, 2016).

Because of the rarity of the complication of GBS, raising awareness among clinicians is likely to be the more effective risk communication strategy. However, the fact that cases may have mild, or no, symptoms of ZIKV infection preceding it means that clinicians won’t necessarily be aided by a clinical prompt, hence delays in diagnosis are likely.

In countries where ZIKV is not active and not receptive to the virus, the focus of awareness-raising is on those citizens planning to travel to Zika-affected areas, in particular, to individuals or couples who are pregnant or planning to become pregnant. Avoiding or deferring travel is advised, and if the person does decide to travel, the messages focus on mosquito avoidance and contraception advice, and symptoms to be aware of on return. Again, the high rate of asymptomatic infection means that the advice needs to relate to anyone traveling to endemic areas, not just those who develop symptoms. Based on available evidence of risk of sexual transmission of ZIKV, the WHO recommends men and women returning from areas where transmission of ZIKV is known to occur to abstain from unprotected sex for at least 6 months upon return to prevent ZIKV infection through sexual transmission and that women who are planning a pregnancy wait at least 6 months before trying to conceive to ensure that possible ZIKV infection has cleared (WHO, 2016b). Disease control agencies from individual countries advise a lesser risk period for women to defer pregnancy following their (or their partner’s) return from Zika-affected areas (CDC, 2017).

A third category includes those countries where ZIKV infection is not endemic but are receptive to the virus as they harbor competent vectors. The key population health messages in these countries relate to raising awareness of the symptoms of ZIKV infection so that cases are ascertained early and disease control measures rapidly instigated. Travelers from these countries to endemic areas need to be especially aware of risk mitigation measures whilst traveling to endemic areas, as well as symptoms of the infection, as they are at risk of triggering an outbreak on their return.

### Disease Control Measures

Disease control methods available for the fight against ZIKV are limited. There is neither an effective vaccine against the virus, nor anti-viral drugs to reduce the viremic period during which a competent vector with hematophagic behavior can amplify the disease. Vector control measures are, therefore, crucial and reduction of contact between hosts and vectors form the basis of disease control strategies worldwide.

Two broad types of complementary vector control strategies can be applied. First, individuals must take personal responsibility for avoiding mosquito bites and, second, government and non-government organizations must implement vector surveillance and control programs at global, national and local levels.

Recently, innovative methods to reduce mosquito populations have been shown great promise in laboratory and field conditions. Examples include the employment of insect sterile techniques (Alphey et al., 2010), introduction of *Wolbachia* strains (Jeffries and Walker, 2016) and genetically modified organisms (Beisel and Boëte, 2013), and these may be introduced in future years as adjuncts to conventional mosquito control programs.

In countries that do not have Zika activity but harbor competent vectors of the disease, disinsection of arriving airplanes is an important consideration. The WHO recommends that these countries undertake a risk assessment, and if it concludes that a disinsection program is indicated, that it should be conducted according to standard WHO recommendations (WHO, 2016a).

#### Risk of Transmission from Blood Transfusion

A study of blood donors during the outbreak in French Polynesia, which found that 3% were positive for ZIKV by PCR while asymptomatic (Musso et al., 2014), has led to concerns about the risk of transmission of ZIKV infection during the transfusion of blood products. The risk is small, however, with only one report of infection caused by transfusion of platelets (Motta et al., 2016).

Some countries require blood donors who have traveled to Zika-affected areas defer their donation for a period after their return or after any sexual contact with a known case (CDNA, 2016). Tests to screen blood donations for ZIKV are available and, despite them not being licensed by the United States Food and Drug Administration (US FDA), it recommends screening all donations in the US and the removal of any positive samples from the blood supply (FDA, 2016).

## Perspectives

### Vaccines

Vaccines for several flaviviruses have been produced during the past decades, some of them being already in the market, such as those for YF, tick-borne encephalitis (TBE), or WNF. These vaccines have been produced using different strategies: inactivated or live-attenuated viruses, recombinant proteins and recombinant subviral particles expressed in different heterologous systems, chimeric backbone viruses, or naked cDNA, among others (Martin-Acebes and Saiz, 2012). Thus, after the explosive spread of ZIKV in the Americas that quickly raised social and health worldwide concern because of its possible association with severe neurological pathologies (Blazquez and Saiz, 2016), it was reasonable to think that similar strategies can be applied to ZIKV (Saiz et al., 2016).

In this way, an alum-adjuvanted whole inactivated ZIKV (ZPIV) vaccine candidate recently showed complete protection against detectable viremia in challenged mice (Larocca et al., 2016) and later on in rhesus macaques (Abbink et al., 2016). On the other hand, although live attenuated vaccines are in widespread use for several viral infections, they are usually contraindicated for pregnant women, and in some instances for children, and therefore, they are not a primary target for ZIKV, even though WHO has reported no evidence of increased adverse pregnancy outcomes when licensed vaccines of this kind have been used (WHO, 2014). Similar issues apply for recombinant heterologous viral vectored vaccines and, thus, none of them have been licensed to date, even though a vaccine candidate that incorporates ZIKV pre-membrane and envelope (prM-E) proteins into a rhesus adenovirus serotype 52 viral vector has recently shown complete protection in challenged monkeys 4 weeks after vaccination with a single dose (Abbink et al., 2016).

On the other hand, until now, and though DNA vaccine technology has been available for many years, no such a human vaccine has been licensed; however, a DNA-ZIKV expressing the full-length ZIKV prM-E proteins induced complete protection against viremia both in mice (Larocca et al., 2016) and rhesus macaques (Abbink et al., 2016). Likewise, a recombinant vaccine ZIKV/JEV prM-E DNA constructs, in which the ZIKV prM signal sequence was replaced with the analogous JEV sequence to improve expression, has shown high levels of protection against viremia in challenged rhesus macaques (Dowd et al., 2016b). Even more, adoptive transfer of purified IgG from mice vaccinated with a ZIKV plasmid DNA vaccine conferred passive protection (Larocca et al., 2016), as did those from mice and rhesus macaques inoculated with an inactivated ZIKV vaccine (Abbink et al., 2016). Similarly, mice treated with a monoclonal antibody against the Domain III of ZIKV-E protein were protected from lethal ZIKV infection (Stettler et al., 2016).

As today, more than 30 vaccine candidates are in active preclinical development, and three have been already approved by the FDA to enter phase I clinical trials (WHO, 2017). NCT02809443 (GLS-5700, Inovio Pharmaceuticals and GeneOne Life Sciences) is a synthetic DNA plasmid vaccine which encodes for the pM-E regions of ZIKV that is being tested in healthy volunteers. This candidate is also in a phase I clinical trial in endemic areas in DENV seropositive adults (NCT02963909). NCT02840487 (VRC-ZKADNA085-00-VP, Vaccine Research Center, NIAID) is also composed of a single closed-circular DNA plasmid that encodes the prM-E proteins from ZIKV. NCT02963909 (NIAID) is an alum adjuvanted ZIKV purified inactivated vaccine (ZPIV) that has entered clinical trial in healthy flavivirus-naïve and flavivirus-primed subjects.

However, due to the characteristics of ZIKV infection, several specific concerns should be kept in mind when developing vaccine candidates against the virus. Thereby, given the possible association of the viral infection with congenital abnormalities (Blazquez and Saiz, 2016), the primary target of the vaccine would be pregnant women and women of childbearing age, although men may also be a target, as ZIKV has been detected in semen pointing to a sexual transmission route. In addition, there are also concerns over the possible interaction of preexisting flavivirus immunity with neutralization and/or enhancement, since several flavivirus cocirculate in the ZIKV endemic areas, including DENV for which an antibody dependent enhancement (ADE) effect has been described. Indeed, the relationships between the immune response to ZIKV and previous DENV infection has been recently demonstrated (Dejnirattisai et al., 2016; Stettler et al., 2016). However, no such ADE effect has been observed in ZIKV infected animal models challenge with WNV (Vázquez-Calvo et al., 2017a). Based on an ecological study, a possible protective effect of YZ fever vaccination has been discussed (De Goes Cavalcanti et al., 2016).

Furthermore, as there is no guarantee that experimental promising results will be reflected in the clinical evaluation of vaccine candidates, and based on the previous experience from the Ebola epidemic, where substantial delays occurred before the stakeholders established the necessary agreements, WHO has launched a target product profile (TPP) describing the preferred and minimal product characteristics for a vaccine targeted to the proposed priority populations (Vannice et al., 2016), which certainly will have to be updated in the coming months once new data are available. In fact, there are tests that take months to be evaluated (stability, neurovirulence, toxicity, etc.), but that should be addressed even under emergency circumstances. Moreover, as noted by Dittmer (2016), besides the technical and ethical aspects, several questions arise regarding ZIKV vaccine campaign implementation that should be considered: Do we need a sophisticated expensive vaccine? Is it economically worthy to develop a vaccine and large-scale clinical trials? Moreover, although vaccines potentially provide powerful tools for the control of viral pathogens, as mentioned before, the development of possible adverse effects derived from ADE of infection of related flavivirus (i.e., DENV) should also be extensively considered before starting large-scale vaccination campaigns.

### Antivirals

As mentioned before, nowadays there are no approved specific antiviral agents against any flavivirus (Menendez-Arias and Richman, 2014), and treatment is generally directed to symptom relief with analgesics and antipyretic. However, in the past months, several drugs have been tested *in vitro* and *in vivo* as antiviral candidates (Saiz and Martin-Acebes, 2017), including the screening of different compounds libraries, such those already approved by the FDA, and the repurposing of drugs already used in clinic for other diseases, many of which are broad spectrum molecules.

For instances, different nucleoside analogs/derivatives that target viral polymerases, such as 2′-C-methylated nucleosides, have shown to inhibit ZIKV multiplication in cell culture (Eyer et al., 2016; Zmurko et al., 2016; Hercik et al., 2017), as did Sofosbuvir and BCX4430 (Bullard-Feibelman et al., 2017; Julander et al., 2017; Sacramento et al., 2017), which also induced greater survival rates in treated experimental immunodeficient mice (Bullard-Feibelman et al., 2017; Julander et al., 2017). Likewise, the pyrimidine synthesis inhibitors NITD008, CID 91632869, finasteride, brequinar, 6-azauridine, gemcitabine, and 5-fluorouracil reduced viral multiplication to different levels (Pascoalino et al., 2016; Adcock et al., 2017; Kuivanen et al., 2017). Even more, NITD008 improved survival rates in treated mice infected with ZIKV (Deng et al., 2016). In addition, by means of a high-throughput screening of over 40,000 compounds, it has been shown that non-peptidic small molecules targeting other viral proteins, such as the N2B-NS3 trypsin-like serine-protease, were capable of inhibiting ZIKV multiplication in cell culture (Lee et al., 2017).

On the other hand, it has been recently reported that passive transfer of human neutralizing antibodies to pregnant mice suppressed ZIKV replication and prevent microcephaly (Sapparapu et al., 2016; Wang et al., 2017), as did a monoclonal antibody against the Domain III of ZIKV-E protein protected against lethal ZIKV infection in a murine model (Stettler et al., 2016). Even more, compounds present in many natural products that also target the viral particle, such as the polyphenols epigallocatechin gallate and delphinidin chloride, also exhibit anti-ZIKV activity, probably through a virucidal effect (Carneiro et al., 2016; Vázquez-Calvo et al., 2017b).

Besides drugs targeting viral components, those directed against cellular factors implicated in the viral life cycle have also been assayed, as they are less prone to induce the emergence of resistant virus. In this line, by screening different libraries and bioactive molecules and by drug repurposing, different inhibitors of ZIKV infection were uncover, including the fusion inhibitors SaliPhe, monesin, and niclosamide (Xu M. et al., 2016; Adcock et al., 2017; Kuivanen et al., 2017), as well as others molecules that affect different cellular pathways, like bortzetomib, sertraline (Barrows et al., 2016), palonosetron (Pascoalino et al., 2016), tenovir-1 (Kuivanen et al., 2017), obatoclax (Rausch et al., 2017), PHA-690509, and emirascan (Xu M. et al., 2016). The immunosuppressants cyclosporine A, mycophenolic acid, and azathiophine have also been tested with promising results (Barrows et al., 2016). Likewise, hypolipidemic drugs like lovastatin, PF-429242, fatostatin, nordihydroguaiaretic acid, and tetra-O methyl nordihydroguaiaretic acid have demonstrated inhibitory activity against ZIKV in cell culture (Pascoalino et al., 2016; Merino-Ramos et al., 2017).

Finally, antiparasitics and antimalarials, such as ivermectin, chloroquine, quinacrine, mefloquine, GSK-36796, and pyrimethamine (Barrows et al., 2016; Delvecchio et al., 2016; Balasubramanian et al., 2017), as well as antibiotics like nanchagmycin, daptomycin and kitasmycin (Barrows et al., 2016; Pascoalino et al., 2016; Rausch et al., 2017) have also been shown to reduce ZIKV multiplication. Nevertheless, and despite the great effort made by the scientific community, it will take time until any drug against ZIKV will be commercially available.

## Author Contributions

All authors listed have made a substantial, direct and intellectual contribution to the work, and approved it for publication.

## Conflict of Interest Statement

The authors declare that the research was conducted in the absence of any commercial or financial relationships that could be construed as a potential conflict of interest.
